# A novel synonymous variant in the NF1 gene disrupting splicing contributes to neurofibromatosis pathogenesis

**DOI:** 10.3389/fgene.2025.1572487

**Published:** 2025-05-09

**Authors:** Tao Lin, Zheyan Chen, Biwen Dong, Haojie Pan, Hai Wang, Xianjue Zheng, Kaixin Chen, Yanan Lai, Chenhui Zhang, Ye Dong, Zitong Xu, Menmen Lin, Xiujie Xi, Shuqi Xia, Yimin Wang, Wenhan Wang, Xiaoqing Li, Congcong Sun, Yanjun Hu, Fang Xu, Jianqiong Zheng, Fan Jin, Hongping Zhang, Jiayong Zheng

**Affiliations:** ^1^ Department of Gynecology and Obstetrics, Wenzhou People’s Hospital/Wenzhou Maternal and Child Healthcare Hospital, Wenzhou, China; ^2^ Department of Plastic and Aesthetic Surgery, Wenzhou People’s Hospital/Wenzhou Maternal and Child Healthcare Hospital, Wenzhou, China; ^3^ Department of Reproductive Genetics, Wenzhou Third Clinical Institute Affiliated to Wenzhou Medical University/The Third Affiliated Hospital of Shanghai University/Wenzhou People’s Hospital/Wenzhou Maternal and Child Healthcare Hospital, Wenzhou, China; ^4^ Wenzhou City Key Laboratory of Gynecology and Obstetrics, Wenzhou, Zhejiang, China; ^5^ Department of Reproductive Genetics, Women’s Hospital, Zhejiang University School of Medicine, Hanzhou, China

**Keywords:** neurofibromatosis type 1, *NF1*, synonymous variation, splicing variant, whole-exome sequencing

## Abstract

**Background:**

Neurofibromatosis type 1 (NF1) is a common autosomal dominant genetic disorder characterized by café-au-lait macules, neurofibromas, and other manifestations. It is caused by variations in the *NF1* gene located on chromosome 17q11.2. The gene’s complexity and extensive variations often lead to misdiagnoses by conventional detection methods, which adverses to effective diagnosis and treatment strategies.

**Case presentation:**

A 26-year-old Chinese woman was admitted to our hospital with multiple café-au-lait spots and cutaneous nodules. She had a family history of NF1, with her mother also showing similar dermatological symptoms. Whole exome sequencing (WES) identified a synonymous variation, NM_001042492.3: c.987A>G (p.K329K), in the *NF1* gene. Although synonymous variations are typically considered non-pathogenic, RNA sequencing (RNA-seq) and minigene analysis revealed that this variation caused the partial loss of exon 9, leading to aberrant splicing. These findings were validated through Sanger sequencing, confirming the genetic alteration and its impact on mRNA splicing.

**Conclusion:**

The case highlights the critical role of synonymous variations in the *NF1* gene that significantly impact splicing and protein function. These findings expand our understanding of *NF1*’s genetic diversity and underscore the importance of comprehensive genetic and RNA analyses to achieve accurate diagnosis and in-depth insight into the molecular underpinnings of NF1.

## 1 Introduction

Neurofibromatosis type 1 (NF1) (OMIM #162200) is a multisystem autosomal dominant disorder that manifests as skin pigment changes and benign neurofibromas (Anderson and Gutmann, 2015). NF1 affects approximately 1 in 1,900–2,500 live births and shortens life expectancy (Evans et al., 2011; Uusitalo et al., 2015). NF1 is caused by variations in the NF1 gene, which encodes neurofibromin, a negative regulator of RAS GTPase activating protein (GAP) (Ballester et al., 1990; Trovó-Marqui and Tajara, 2006). The NF1 gene, located at 17q11.2, spans approximately 350 kb of genomic DNA and contains 60 exons (Bergoug et al., 2020). It has one of the highest variation rates among human genes, with about 50% of NF1 cases occurring sporadically (Ars et al., 2003).

The HGMD Database (Professional v2023.3) currently catalogs almost 3,890 DNA variants of the NF1 gene, with about half being *de novo* and potentially resulting in mosaic phenotypes (Kehrer-Sawatzki and Cooper, 2008). Most pathogenic variations lead to truncated neurofibromin proteins, and 30% affect mRNA processing (Koster et al., 2021; Xu et al., 2022). These splicing variants disrupt the 3′ or 5′ splice site, leading to exon skipping and intron retention, resulting in abnormal neurofibromin production (Ward and Cooper, 2010; Jayasinghe et al., 2018; Kim et al., 2020). The large size, pseudogenes, and dispersed variations make the challenge to identify NF1 gene variations using conventional genetic testing methods (Cummings et al., 1996). However, RNA-seq delivers an in-depth analysis of the transcriptome, enabling the discovery of abnormal splicing events, changes in gene expression, and novel transcripts, effectively overcoming some of the traditional sequencing methods’ limitations (Koster et al., 2021). Additionally, though traditionally considered non-pathogenic, synonymous variations can cause abnormal splicing, contributing to the disease (Zhou et al., 2020; Knapp et al., 2021; Niersch et al., 2021).

Herein, we present a case involving a 26-year-old Chinese woman with typical NF1 characteristics. Whole exome sequencing (WES) revealed a synonymous variation, NM_001042492.3 c.987A>G (p.K329K), which RNA-seq analysis confirmed resulted in partial loss of exon 9.

## 2 Materials and methods

### 2.1 Patients

The patient and the patient’s mother provided written informed consent for relevant clinical and molecular data in this paper, including histological tumor type, diagnosis, relevant images, and sequencing data. The study was conducted according to the principles outlined in the Declaration of Helsinki, and the Clinical Medical Ethics Committee of Wenzhou People’s Hospital, approved the study’s design.

### 2.2 Whole exome sequencing

The patient’s genomic DNA was extracted from peripheral blood using the nucleic acid extraction kit (AmCare Genomics Lab, Guangzhou, China; hereafter referred to as AmCare) and was fragmented using an enzyme kit (AmCare). The libraries were prepared using the Gene Sequencing Library Kit (AmCare). The whole-exomes were captured using the hybrid capture kit (AmCare), and then adaptors were added. Captured DNA samples were amplified with pre-capture ligation-mediated PCR (LM-PCR) and then sequenced on an AmCare Seq-2000 sequencer (AmCare). The raw reads were processed using fastp to remove adapters, polyN, polyA, and low-quality reads (QC < 20), then mapped to the hg19 reference genome using Burrows-Wheeler Aligner (BWA, v0.7.15). The average sequencing depth of the target region is ≥200×, and more than 96% of the target regions are covered by at least 20 reads.

### 2.3 Bioinformatic analysis

All variants were identified using an in-house bioinformatics pipeline (AmCare), which includes proprietary algorithms for extracting variant sites of single nucleotide polymorphisms (SNPs) and GATK’s HaplotypeCaller for insertions and deletions (InDels), followed by filtering with VQSR. Variants with a minor allele frequency (MAF) greater than 1% in the Asian population and those with a variant allele frequency (VAF) below 15% were excluded. Pathogenicity was assessed using databases such as the Genome Aggregation Database (GnomAD; http://gnomad.broadinstitute.org), ClinVar (https://www.ncbi.nlm.nih.gov/clinvar/), OMIM, peer-reviewed literature. Variants were classified into five categories: Pathogenic, Likely Pathogenic, Variant of Uncertain Significance (VUS), Likely Benign and Benign according to ACMG/AMP guidelines (Richards et al., 2015). AmCare laboratory further employs an internal sub-tier classification for VUS, subdividing them as VUS-A (higher pathogenic likelihood), VUS-B (moderate), or VUS-C (lower), to facilitate clinical counseling.

### 2.4 Sanger validation

The candidate variants were amplified by PCR and sequenced by Sanger sequencing in an ABI 3130-xl Genetic Analyzer (Applied Biosystems, Foster City, CA, USA). All primers are available in Supplementary Table S1. The sequences were aligned with a reference sequence from NCBI (http://www.ncbi.nlm.nih.gov) to confirm candidate nucleotide changes. To determine sequence variability, variable sites in patient genes were aligned with the corresponding sites from the mother.

### 2.5 RNA sequencing

Peripheral blood from the patient was collected into a PAXgene tube, and total RNA was extracted according to the standard operating instructions for the nucleic acid extraction kit (AmCare). The RNA was then reverse-transcribed into complementary DNA (cDNA) using the ReverseScript RNA kit (AmCare). The cDNA was subsequently subjected to RNA sequencing (RNA-seq), which included DNA fragmentation, end repair, adapter ligation, and PCR amplification. These steps were conducted using protocols similar to those used in WES. The prepared libraries were sequenced on an AmCare Seq-2000 sequencer to assess gene expression.

### 2.6 Long-range polymerase chain reaction RNA-seq

The mRNA extracted from the patient’s blood was reverse-transcribed into cDNA using random primers. The resulting cDNA was then amplified using PCR. Following PCR amplification, the cDNA was further subjected to RNA-seq to minimize the risk of homologous interference and ensure reliable identification and analysis of genetic variation.

### 2.7 Gene acquisition and recombinant vector construction

Two pairs of nested primers were designed by Primer 3 software, and nested PCR was performed using genomic DNA as a template. Two rounds of nested PCR products were used as templates, and pcMINI-NF1-KpnI-F and pcMINI-NF1-XhoI-R, pcMINI-C-NF1-KpnI-F and pcMINI-C-NF1-XhoI-R were used as primers to obtain pcMINI (1,138 bp) and pcMINI-C (1,230 bp), respectively. The wild-type and mutant fragments containing the restriction endonuclease cleavage site of NF1 gene were obtained. Four recombinant vectors were obtained by inserting wild-type and mutant minigenes into pcMINI and pcMINI-C vectors, respectively. The recombinant plasmid was obtained by transformation, colony PCR identification and sequencing.

### 2.8 Cell transfection and minigene analysis

HeLa and 293T cells were cultured in DMEM medium containing 10% fetal bovine serum, and the four recombinant plasmids were transfected into HeLa and 293T cells by liposome transfection method. After 48 h of transfection, 8 samples were collected and RNA was extracted with TRIzol reagent. After reverse transcription, PCR amplification was performed using vector flanking primers, and Sanger sequencing was used to verify whether there was abnormal splicing.

## 3 Results

### 3.1 Clinical features

**FIGURE 1:**
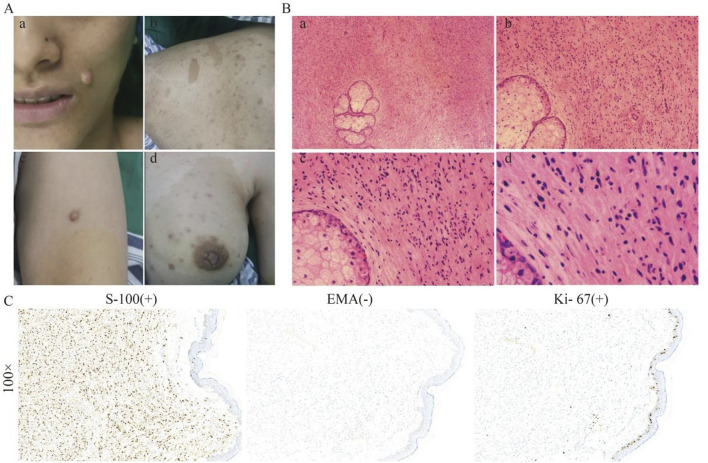
Clinical and histological features of the proband. **(A)** The proband showed CALMs, cNFs, and Lisch nodules on her face, trunk, and breast. **(B)** H&E staining of a skin biopsy at different magnifications, showing the general architecture of the skin tissue, including fibroblasts and collagen fibres, increased cellularity, fibroblast proliferation within the dermis, and spindle-shaped cells. **(C)** IHC staining at ×100 magnification for S-100, showing strong positivity, indicating the presence of neural crest-derived cells; for EMA, showing negative staining, ruling out epithelial origin; and for Ki67, showing low expression, indicating minimal cellular proliferation.

The proband, a 26-year-old Han Chinese female, was admitted to our department on 11 April, 2023, and diagnosed with NF1. She had experienced multiple Café-au-lait macules (CALMs) on her face, trunk, and limbs, and cutaneous nodules for over 20 years (Figure 1A). Hematoxylin and eosin (H&E) staining revealed that the tumor cells were elongated and spindle-shaped, with some showing ovoid nuclei. The chromatin was finely dispersed, nuclear borders were well-defined, and no significant mitotic was observed (Figure 1B). Immunohistochemical analysis (IHC) demonstrated that the tumor cells were S-100 protein (S-100) positive, epithelial membrane antigen (EMA) negative, and Ki-67 antigen weakly positive, suggesting a neurogenic origin with low proliferative activity (Figure 1C). Microscopic examination of the blood vessels revealed structurally typical vessels without significant abnormalities, indicating no apparent vascular involvement by NF1. Similarly, the sweat glands appeared normal in morphology and distribution, without hypertrophy, atrophy, or dysplasia (Supplementary Figure 1A). Although the proband reported mild hearing loss, an audiological examination showed no abnormalities. She also had reduced vision in the left eye and had a history of constipation and otitis media. Routine blood tests indicated an elevated eosinophil percentage (8.7%), high total cholesterol (5.32 mmol/L), and decreased serum albumin (8.7%). Ultrasound examinations revealed hepatic echogenicity, hemangiomas within the liver, and gallbladder masses measuring up to 5 mm. Pulmonary CT scans identified minute nodules. The electrocardiogram indicated sinus tachycardia. The proband showed slight cranial depression, and a CT scan in 2023 revealed high-density shadows in the bilateral semioval centers and thickened cranial bones (Supplementary Figure 1B). However, no further examinations were conducted. The proband was initially clinically diagnosed with non-malignant NF1.

**FIGURE 2:**
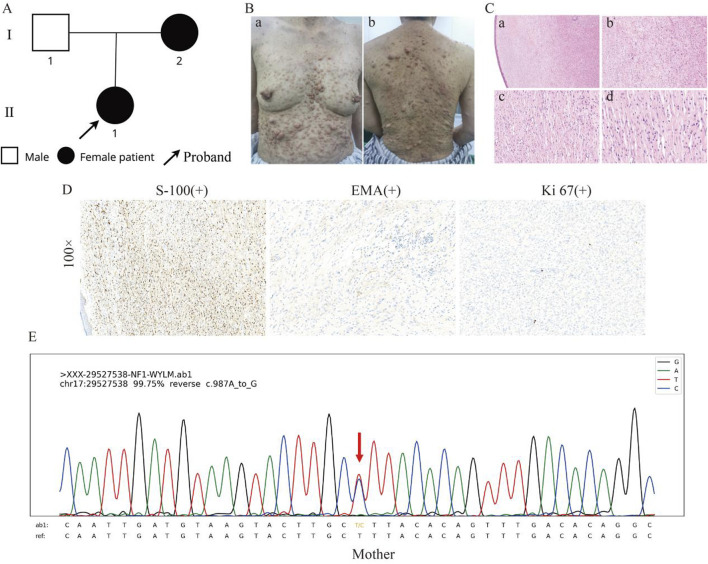
Clinical and histological features of the proband’s mother. **(A)** Family pedigree showing the proband (II-1) and her affected mother (I-2). **(B)** The clinical presentation of the proband’s mother, displaying more pronounced CALMs and cutaneous nodules than the proband. **(C)** HE staining of the skin at different magnifications, showing the general architecture of the skin tissue, including fibroblasts and collagen fibres, increased cellularity, fibroblast proliferation within the dermis, and spindle-shaped cells. **(D)** IHC results at ×100 magnification, with S-100 showing positive staining, indicating the presence of neural crest-derived cells; EMA showing positive staining, indicating epithelial origin; and Ki67 showing low expression, indicating minimal cellular proliferation. **(E)** Sanger sequencing results show the presence of the c.987A>G variant in the proband’s mother.

In this pedigree, the proband (II-1) showed characteristic symptoms of NF1. The proband’s mother (I-2) is also affected (Figure 2A). The proband’s mother, a 53-year-old Han Chinese female, had experienced similar cutaneous manifestations, presenting multisite neurofibromas, more pronounced CALMs, and cutaneous nodules than the proband (Figure 2B). H&E staining of skin biopsy was the similar as the proband (Figure 2C). IHC staining was positive for S-100, focal EMA, with Ki67 expression less than 1% (Figure 2D). Microscopic examination of the sebaceous glands revealed typical lobular architecture with clusters of sebocytes, without hyperplasia or neoplasia. Blood vessels appeared structurally normal without significant dilation or thrombosis. Sweat glands and ducts showed typical morphology without hypertrophy, atrophy, or dysplasia (Supplementary Figure 1C). Her mother reported anemia. Upon Routine blood examination, the test results indicated low red blood cell mean corpuscular volume (81.3 fL), mean erythrocyte hemoglobin volume (25.8 pg), mean platelet volume (8.5 fL), monocyte percentage (0.026%), and absolute monocyte count (0.09*10^9^/L), with high platelet distribution width (15.2 fL). Pulmonary CT scans suggested the presence of a malignant tumor. Her mother experienced a sudden onset of cerebral epilepsy during chemotherapy, but no further examinations were performed. Her mother was also initially clinically diagnosed with non-malignant NF1. The proband’s father, a 57-year-old Han Chinese male, had a history of vitiligo and hepatitis, with no other apparent abnormalities.

### 3.2 Genetic analysis

A heterozygous synonymous variant (NM_001042492.3: c.987A>G, p. K329K) was identified in the *NF1* gene of the proband. The p. K329K variant is recorded and categorized as VUS in the ClinVar database without a detailed report. However, it is not recorded in the GnomAD Database. This variant p. K329K was predicted to have a low likelihood of affecting the splicing function by Alamu. Following ACMG guidelines, the variant p. K329K is classified as VUS (C). Sanger sequencing confirmed the presence of this variant in the proband’s mother (Figure 2E). Based on the clinical and genetic characteristics, this indicates an inherited variant.

### 3.3 RNA splicing analysis

**FIGURE 3:**
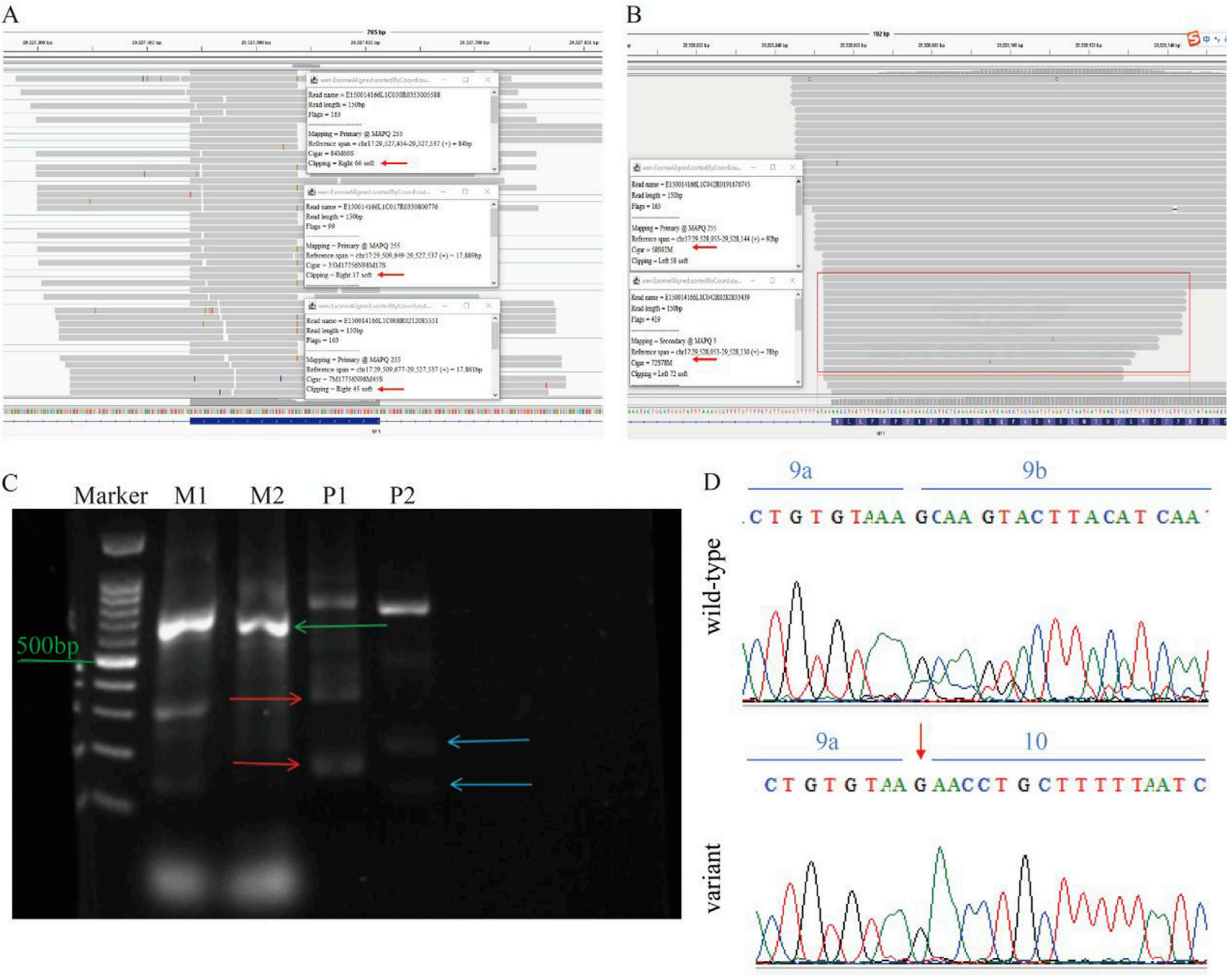
mRNA Analysis of the NF1 Gene. **(A)** RNA-seq data shows partial bases deletion to the right of the variation site in exon 9 of the proband’s NF1 gene. **(B)** RNA-seq data indicating partial deletion of bases on the left side of some reads in exon 10. **(C)** Gel electrophoresis results show two bands corresponding to the expected lengths for the wild-type and the mutated allele, with a 75 nucleotide deletion observed. M represents the mother, and P represents the proband. **(D)** Sanger sequencing of cDNA confirms the heterozygous variation at position c.987A>G within exon 9, revealing partial loss of exon 9.

RNA-seq revealed that in the proband’s *NF1* gene, there was a partial deletion of bases to the right of the variation site in exon 9 and on the left side of some reads of exon 10 (Figures 3A,B). Gel electrophoresis revealed two bands with sizes corresponding to the expected lengths for both the wild-type and the mutated allele. Specifically, for Primer pair 1, the proband carrying the heterozygous p. K329K variant displayed two PCR products, measuring 221 and 155 nucleotides, representing the wild-type and the mutated allele, respectively. Similarly, Primer pair 2 displayed two PCR products of 186 and 111 nucleotides, aligning with the wild-type and mutated alleles, respectively (Figure 3C). This result showed a consistent 75 nucleotide deletion between the bands for both primer pairs. Sanger sequencing validated a heterozygous variation at position c.987A>G within exon 9 was identified (Figure 3D). This variation leads to a synonymous change, p. K329K, without altering the amino acid sequence.

### 3.4 LR-PCR RNA-seq analysis

**FIGURE 4:**
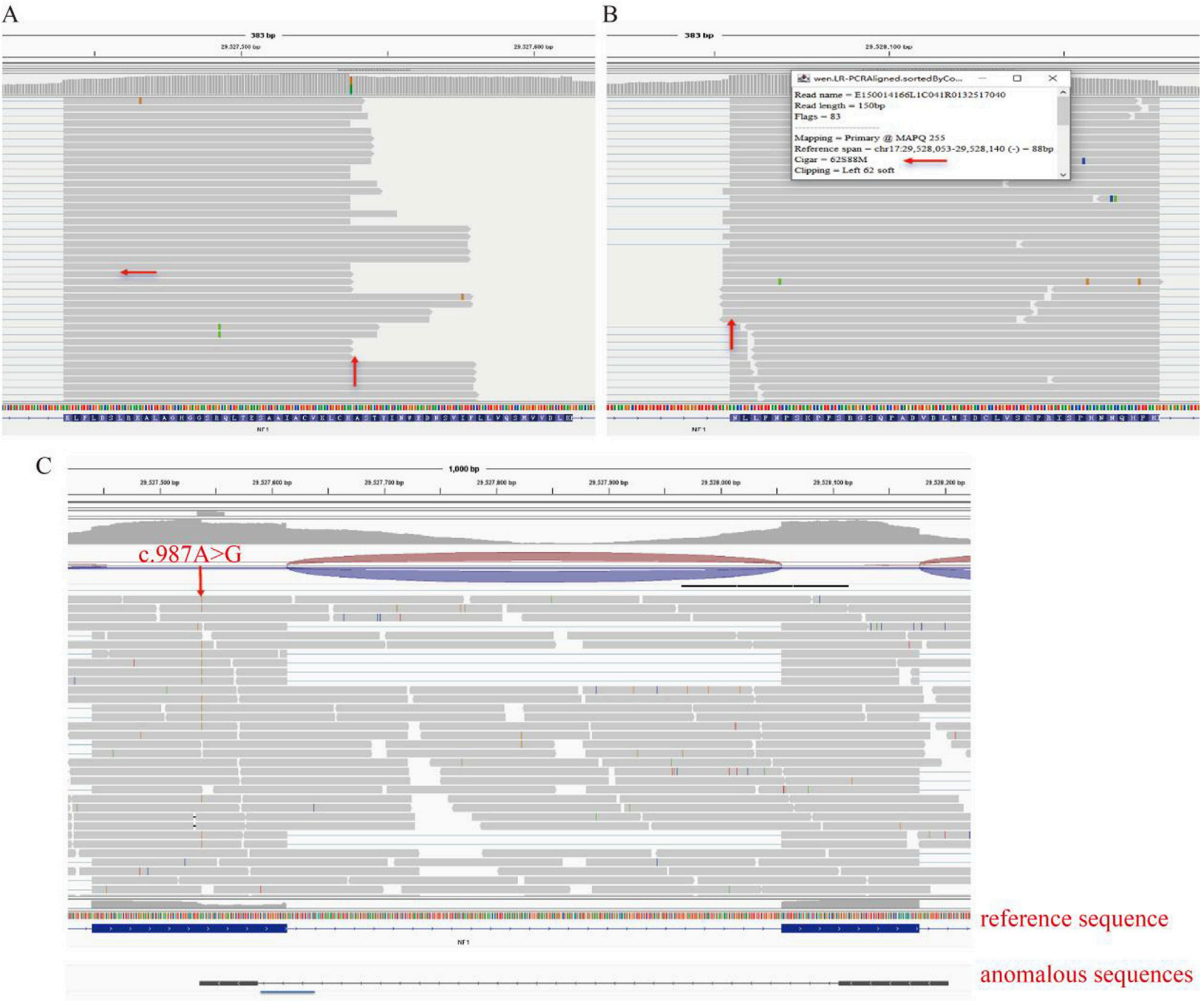
Long-Range PCR RNA-seq Analysis. **(A)** Long-range PCR RNA-seq data shows a loss of partial read bases in exon 9 of the NF1 gene. **(B)** Long-range PCR RNA-seq data showing a loss of partial read bases in exon 10 of the NF1 gene. **(C)** Aligning RNA-seq sequences with the reference genome highlights the consistent lack of expression of a segment of the exon post-variation site.

Long-range PCR (LR-PCR) revealed a loss of partial read bases in both exon 9 and exon 10 of the *NF1* gene. Specifically, there was a noticeable absence of sequence reads to the right of the variation site in exon 9 and the left in some reads of exon 10 (Figures 4A,B). This pattern suggests a deletion event affecting the RNA transcript. Aligning the obtained sequences from RNA-seq with the reference genome highlighted a consistent lack of expression of a segment of the exon post-variation site (Figure 4C).

### 3.5 Mini-genen analysis

**FIGURE 5:**
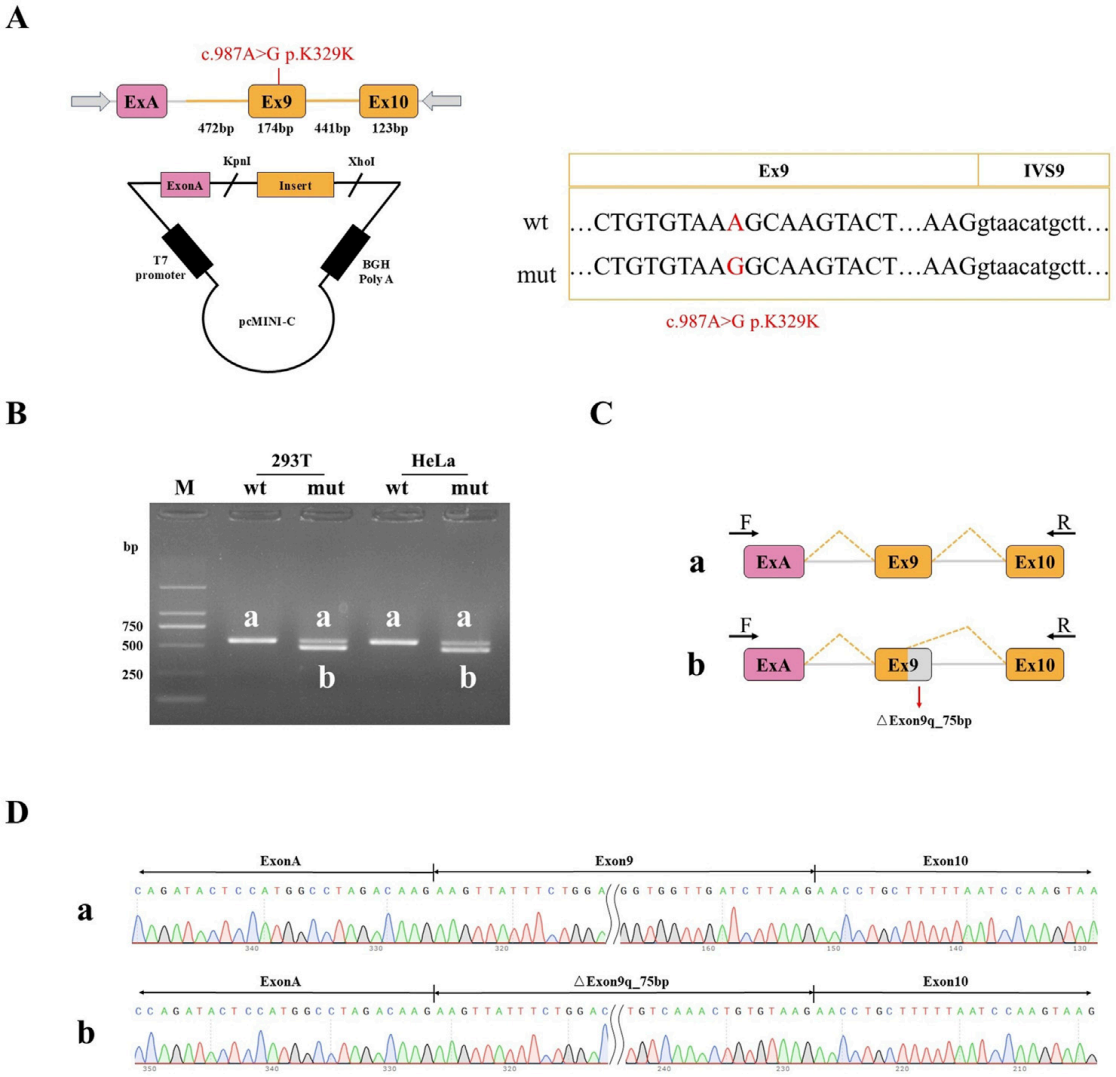
Minigene detection results **(A)** pcMINI-C vector construction strategy map; **(B)** RT-PCR transcription analysis of agarose gel electrophoresis; in HeLa and 293 T cells, the bands were labeled as **(a,b)**; **(C)** minigene cutting diagram; **(D)** The corresponding sequencing results of the shear bands.

Part of the intron8 (472bp) -Exon9 (174bp) -intron9 (441bp) -Exon10 (123bp) was inserted into the pcMINI-C vector, and the cells were transfected to observe whether the shear mode of ExonA-Exon9-Exon10 was abnormal (Figure 5A). The results of agarose gel electrophoresis after RT-PCR showed that the wild type was a single band in HeLa and 293T cells, which was basically consistent with the expected size (557bp), and was named as band a. The wild type band a in the 2 cell lines was purified and directly subjected to Sanger sequencing. The mutant was divided into two bands in HeLa and 293T cells, of which the large band was named band a and the small band was named band b. The mutant bands in the 2 cell lines were purified and TA cloned for Sanger sequencing. The results showed that the mutant band a was a normal shear band, the mutant band b was an abnormal shear band, and the abnormal condition was a 75 bp deletion on the right side of Exon9 (Figures 5B–D). In addition, the detection results of the recombinant plasmids of pcMINI series constructed by us were consistent with those of pcMINI-C, and the mutant type also had normal shear band a and abnormal shear band b.

The mutation resulted in the deletion of 75bp on the right side of Exon9, and the expression pattern at the cDNA and protein levels was c.988 _ 1062del p. Ala330 _ Lys354del. The deletion of 75 bp on the right side of Exon9 did not lead to subsequent changes in the reading frame, but only resulted in the deletion of 25 aa in the protein, which may produce a truncated protein with a length of 2,814 aa.

### 3.6 ACMG classification update for *NF1* variant

The *NF1* variant (NM_001042492.3: c.987A>G, p. K329K) was initially classified as VUS-C based on the evidence with PM2_P (total variant frequency in the population as zero) and PP3_C3 (the relatively low likelihood of this variant impacting splicing function predicted by multiple algorithms). Following comprehensive analyses, including RNA-seq, LR-PCR RNA-seq, and Sanger sequencing, which revealed a 75-base deletion affecting splicing functionality and resulting in protein length changes, the ACMG classification was re-evaluated. The variant is now upgraded to VUS-A based on updated evidence with PM2_P, PP3_A1 (*highly conserved genomic region*), PVS1 (significant impact on splicing indicated by multiple algorithms), PM4 (*in-frame deletion altering protein length*), and PP4 (*clinical presentation consistent with NF1*).

## 4 Discussion

In this study, we identified a rare synonymous variation c.987A>G (p.K329K) in the *NF1* gene, which disrupts splicing and leads to partial loss of exon 9, resulting in aberrant splicing. This is only the second reported synonymous variation in NF1 that disrupts normal splicing, the first being c.6795C>T (p.N2265N) (Jin et al., 2021). Our findings broaden the current understanding of *NF1* gene variations that can disrupt splicing and protein function, challenging the previous notion that synonymous variations are non-pathogenic (Sauna and Kimchi-Sarfaty, 2011; Liu et al., 2020). This discovery emphasizes the importance of exploring the functional impact of synonymous variations, which may alter gene expression and splicing regulatory mechanisms, even without affecting the amino acid sequence.

Synonymous variations have traditionally been overlooked in genetic analysis due to their lack of impact on the amino acid sequence of encoded proteins. However, accumulating evidence suggests that synonymous variations can significantly influence gene expression and function by disrupting splicing regulatory sites, impacting mRNA stability, miRNA binding, or altering translation efficiency (Sharp, 2022; Zhang and Bebok, 2022). Our identification of the p. K329K variation leading to partial loss of exon 9 is consistent with these findings, further highlighting the critical need to consider synonymous changes as potential contributors to genetic diseases like NF1.

The proband and her mother both presented with the same synonymous variation (p.K329K), yet showed distinct clinical manifestations. The proband showed more extensive systemic symptoms, such as sinus tachycardia, liver hemangiomas, gallbladder masses, lung nodules, bilateral semioval center shadows, and thickened cranial bones. On the other hand, her mother primarily experienced anemia and a malignant lung tumor. The differences in clinical phenotypes between these two individuals underscore the significant phenotypic heterogeneity characteristic of NF1, even among patients carrying the same variant. This observation is consistent with the high variability reported for NF1 patients in the literature (Anderson and Gutmann, 2015). Among the patients reported in the literature, some patients (such as patient pairs (#5) and (#6), (#13) and (#19), (#41) and (#42), and (#43) and (#44)) with the same variants displayed similar but not identical symptoms. These findings illustrate the complex interplay between genetic background, modifier genes, and environmental factors in determining the ultimate clinical manifestations of NF1.

In our study, both the proband and her mother showed classical NF1 manifestations, including multiple café-au-lait macules (CALMs), cutaneous neurofibromas (cNFs), and Lisch nodules. To further explore how different splicing variants in *NF1* may lead to diverse phenotypes, we performed a targeted literature review from 2019 to 2024. Only cases with sufficient clinical data, a confirmed NF1 diagnosis, and available evidence of splice alteration were included (Table 1). Our findings are consistent with prior studies, where the most commonly reported clinical features in NF1 patients include CALMs, freckling, cNFs, and Lisch nodules (Table 1) (Kang et al., 2020; Scala et al., 2021; Douben et al., 2022; Wu et al., 2022; Srivastava et al., 2024). Specifically, 43 patients presented CALMs, 27 patients showed freckling, 37 patients had cNFs, 20 patients presented Lisch nodules, 18 patients developed tumors, 17 patients had short stature, 14 patients showed plexiform neurofibromas (PNs), and 14 patients showed scoliosis. Notably, 13 patients (patients #4, #9, #12, #13, #15, #17, #28, #29, #30, #32, #41, #43, #46) showed a combination of CALMs, freckling, cNFs, and Lisch nodules. This extensive phenotypic characterization underlines the variability in the expression of NF1, where even identical mutations can lead to diverse clinical presentations.

**TABLE 1:** Summary of identified genetic variants and clinical findings of the patients.

#Patients	Sex	Age	Identified variation			Clinical findings						
			Gene	c	p	CALMs	Freckling	cNF	PN	Lisch nodules	Scoliosis	Other findings
**#**1 this study	F	26 years	*NF1*	c.987A>G	p.K329K	Y	N	Y	N	Y	N	S-100 (+), Ki-67 (+), EMA (−); eosinophil percentage↑, TC↑, ALB↓; hepatic echogenicity, liver hemangiomas, gallbladder masses (up to 5 mm); minute lung nodules; sinus tachycardia; left eye vision reduction; history of constipation and otitis media; Bilateral semioval center shadows and thickened cranial bones on brain CT
**#**2 this study	F	53 years	*NF1*	c.987A>G	p.K329K	Y	N	Y	N	Y	N	S-100 (+), CK(+), SMA (+), CD68 (+), focal EMA (+), Ki67(<1%); anemia, RBC↓ MCV↓, MCH↓, MPV↓, monocyte percentage↓, absolute monocyte count↓, PDW↑; Malignant lung tumor on CT
**#**3 Kang, 2020 (pt#46)	M	39 years	*NF1*	c.6580–2A>G	IVS42(-2)A>G	Y	N.A.	Y	N	Y	Y	short stature; Nerve root tumor
**#**4 Kang, 2020 (pt#68)	M	27 years	*NF1*	c.3113 + 1G>A	IVS23(+1)G>A	Y	Y	Y	Y	Y	N	short stature; Nerve root tumor; Dysplasia of long bone
**#**5 Kang, 2020 (pt#88)	F	50 years	*NF1*	c.4662–2A>G	IVS34(-2)A>G	Y	Y	Y	N	N.A.	Y	short stature; hearing impairment; Dural ectasia
**#**6 Kang, 2020 (pt#89)	M	18 years	*NF1*	c.4662–2A>G	IVS34(-2)A>G	Y	N	Y	Y	N.A.	Y	short stature; Neuroimaging abnormalities
**#**7 Kang, 2020 (pt#110)	M	36 years	*NF1*	c.6859–2A>C	IVS45(-2)A>C	Y	Y	Y	N.A.	N.A.	Y	short stature; Hypertension; Nerve root tumor; Dysplasia of long bone
**#**8 Kang, 2020 (pt#116)	F	26 years	*NF1*	c.4514 + 1G>A	IVS33(+1)G>A	Y	Y	Y	N	N	Y	short stature; Hypertension
**#**9 Kang, 2020 (pt#151)	F	35 years	*NF1*	c.6364 + 1G>A	IVS41(+1)G>A	Y	Y	Y	Y	Y	Y	short stature; Nerve root tumor; Malignant peripheral nerve sheath tumor
**#**10 Kang, 2020 (pt#157)	M	19 years	*NF1*	c.3975–2A>G	IVS29(-2)A>G	Y	N.A.	Y	Y	Y	N	short stature; Brain tumor
**#**11 Kang, 2020 (pt#165)	M	22 years	*NF1*	c.3113_1G>A	IVS23(+1)G>A	Y	N	Y	Y	Y	Y	short stature; Hypertension; Nerve root tumor
**#**12 Kang, 2020 (pt#195)	F	37 years	*NF1*	c.60 + 1G>A	IVS1(+1)G>A	Y	Y	Y	N.A.	Y	N.A.	Relative macrocephaly
**#**13 Kang, 2020 (pt#219)	M	18 years	*NF1*	c.7395–2A>G	IVS49(-2)A>G	Y	Y	Y	N	Y	Y	short stature; Neuroimaging abnormalities
**#**14 Kang, 2020 (pt#234)	F	18 years	*NF1*	c.3975-1G>C	IVS29(-1)G>C	Y	N.A.	Y	Y	N.A.	Y	short stature; Neuroimaging abnormalities; Dysplasia of long bone; Dural ectasia
**#**15 Kang, 2020 (pt#239)	M	30 years	*NF1*	c.7258 + 1G>C	IVS48(+1)G>C	Y	Y	Y	Y	Y	N	short stature; Other skeletal alterations (e.g., osteoporosis, vertebral defects, *etc.*)
**#**16 Kang, 2020 (pt#240)	F	33 years	*NF1*	c.7258 + 1G>C	IVS48(+1)G>C	Y	N	N	N.A.	N.A.	N.A.	N
**#**17 Kang, 2020 (pt#260)	F	30 years	*NF1*	c.7126 + 1G>A	IVS47(+1)G>A	Y	Y	Y	Y	Y	N	short stature
**#**18 Kang, 2020 (pt#300)	F	30 years	*NF1*	c.2251 + 2T>G	IVS18(+2)T>G	Y	Y	Y	N.A.	N.A.	N.A.	short stature
**#**19 Kang, 2020 (pt#307)	M	20 years	*NF1*	c.7395–2A>G	IVS49(-2)A>G	Y	Y	Y	N.A.	N.A.	N.A.	Relative macrocephaly; short stature
**#**20 Kang, 2020 (pt#323)	F	38 years	*NF1*	c.1721 + 3A>G	IVS15(+3)A>G	Y	Y	N	N	N.A.	N	Relative macrocephaly; short stature; Dysplasia of long bone
**#**21 Scala, 2021 (pt#16)	F	50 years	*NF1*	c.288 + 2T>C	—	Y	Y	Y	N	N	N	Neuroimaging abnormalities
**#**22 Scala, 2021 (pt#17)	F	33 years	*NF1*	c.288 + 5G>A	—	Y	N	Y	N	N	N	Optic pathway glioma
**#**23 Scala, 2021 (pt#42)	F	18 years	*NF1*	c.586 + 1G>C	—	Y	N	N	N	N	N	Optic pathway glioma
**#**24 Scala, 2021 (pt#43)	F	43 years	*NF1*	c.587–3C>G	—	Y	N	Y	Y	N	N	Behavioural abnormalities (ADHD, ASD, ARFID)
**#**25 Scala, 2021 (pt#63)	F	18 years	*NF1*	c.1062 + 1G>T	—	N	N	N	N	N	N	Optic pathway glioma
**#**26 Scala, 2021 (pt#73)	F	20 years	*NF1*	c.1260 + 1G>T	—	Y	Y	N	N	Y	N	N.A.
**#**27 Scala, 2021 (pt#86)	M	19 years	*NF1*	c.1527 + 1G>T	—	Y	Y	N	N	N	N	Optic pathway glioma
**#**28 Scala, 2021 (pt#97)	M	20 years	*NF1*	c.1721 + 3A>G	—	Y	Y	Y	Y	Y	N	N
**#**29 Scala, 2021 (pt#126)	F	37 years	*NF1*	c.2409 + 1_2409+2insCCC	—	Y	Y	Y	Y	Y	N	Neuroimaging abnormalities
**#**30 Scala, 2021 (pt#148)	M	66 years	*NF1*	c.2991–2A>T	—	Y	N	Y	N	N	N	N
**#**31 Scala, 2021 (pt#153)	M	18 years	*NF1*	c.3198–2A>G	—	Y	Y	Y	N	N	N	Other skeletal alterations (e.g., osteoporosis, vertebral defects, etc.)
**#**32 Scala, 2021 (pt#171)	F	18 years	*NF1*	c.3708 + 1G>T	—	Y	Y	Y	Y	Y	Y	N
**#**33 Scala, 2021 (pt#226)	F	37 years	*NF1*	c.5268 + 1G>C	—	Y	Y	Y	Y	N	N	N
**#**34 Scala, 2021 (pt#246)	F	66 years	*NF1*	c.5610-1G>A	—	Y	N	Y	N	N	N	N
**#**35 Scala, 2021 (pt#268)	F	20 years	*NF1*	c.6427 + 2T>A	—	Y	N	Y	Y	N	Y	N
**#**36 Scala, 2021 (pt#272)	M	19 years	*NF1*	c.6642G>A	—	Y	N	N	N	Y	Y	Gliomas other than OPG (e.g., pilocytic astrocytoma, glioblastoma, *etc.*)
**#**37 Scala, 2021 (pt#273)	F	42 years	*NF1*	c.6643-1G>A	—	Y	N	Y	N	N	N	Optic pathway glioma; Gliomas other than OPG (e.g., pilocytic astrocytoma, glioblastoma, *etc.*)
**#**38 Scala, 2021 (pt#275)	M	19 years	*NF1*	c.6704 + 1G>T	—	N	N	N	N.A.	N	N	Optic pathway glioma
**#**39 Scala, 2021 (pt#324)	M	31 years	*NF1*	c.7970 + 5G>A	—	Y	Y	Y	N	N	Y	Brain tumor
**#**40 Srivastava, 2024 (pt#17)	F	18 years	*NF1*	c.4773–2 A>G	—	Y	N.A.	N.A.	N.A.	N.A.	N.A.	short stature
**#**41 Wu, 2022 (pt#2)	M	31 years	*NF1*	c. 4836-10T > G	—	Y	Y	Y	N	Y	N	N
**#**42 Wu, 2022 (pt#3)	F	53 years	*NF1*	c. 4836-10T > G	—	N	Y	Y	N	Y	N	N
**#**43 Douben, 2022 (pt#2)		30 years	*NF1*	c.888 + 789A>G	—	Y	Y	Y	N	Y	N	N
**#**44 Douben, 2022 (pt#4)		34 years	*NF1*	c.888 + 789A>G	—	Y	Y	Y	N	N.A.	Y	Optic pathway glioma
**#**45 Douben, 2022 (pt#6)		36 years	*NF1*	c.5749 + 267A>G	—	Y	Y	Y	N	N	N	Optic pathway glioma
**#**46 Douben, 2022 (pt#7)		22 years	*NF1*	c.1722–26T>G	—	Y	Y	Y	N	Y	N	N

ALB, serum albumin, CALMs, Café-au-lait macules, cNF, cutaneous neurofibromas, IHC, immunohistochemical staining, MCH, mean corpuscular hemoglobin, MPV, mean platelet volume, N, no, N.A., not available, Pt, patient, PN, plexiform neurofibromas, RBC MCV, PDW, platelet distribution width, Red Blood Cell Mean Corpuscular Volume, TC, total cholesterol, yr, years, Y, yes.

The RNA-seq analysis in our study revealed that the synonymous variation c.987A>G (p.K329K) in the *NF1* gene leads to partial loss of exon 9, demonstrating a direct impact on splicing. Previous studies suggested that splicing disruption can occur even without alterations at canonical splicing sites (Zhou et al., 2020). In this case, the synonymous change likely affects splicing regulatory elements (SREs), such as exon splicing enhancers (ESEs) or exon splicing silencers (ESSs), thus disrupting the normal splicing machinery (Tubeuf et al., 2020). ESEs and ESSs play a key role in modulating RNA splicing by attracting or repelling specific splicing factors, thereby regulating the inclusion or exclusion of exons during mRNA processing (Sahebi et al., 2016; Wang and Cooper, 2007; Czech et al., 2010; Jobbins et al., 2018). Our study aligns with previous findings indicating that even synonymous variations can influence the splicing process by modifying the interaction of splicing factors with SREs (Cartegni et al., 2002; Chamary et al., 2006; Pagani et al., 2005).

In this context, splicing regulation plays a critical role in gene expression. Normally, alternative splicing (AS) is a key mechanism that produces multiple protein isoforms from a single gene, contributing to the diversity and adaptability of the proteome. However, our study demonstrates that the p. K329K synonymous variant in the *NF1* gene disrupts normal splicing, causing a partial (75 bp) loss of exon 9 rather than a natural AS event, and leading to a potentially pathogenic outcome. Although AS events, such as exon skipping, intron retention, and the use of alternative splice sites, enrich the functional complexity of eukaryotic proteins (Ramanouskaya and Grinev, 2017), the aberrant event described here most likely arises from the disruption of splicing regulatory elements by this specific genetic change. Similar disruptions in *NF1* caused by other synonymous variants can also lead to significant phenotypic consequences, as evidenced in our proband with multiple systemic symptoms. Our findings underscore the importance of functional splicing analyses, such as RNA sequencing, to detect pathogenic splicing abnormalities that may be overlooked by conventional DNA-based sequencing methods (Mort et al., 2014; Jayasinghe et al., 2018; Kim et al., 2020). By distinguishing natural physiological alternative splicing from mutation-induced aberrant splicing, we gain deeper insights into the molecular basis of disease and provide a clearer framework for understanding the pathogenic mechanisms underlying NF1.

Long-range PCR (LR-PCR) RNA-seq provided additional evidence supporting partial loss of exon 9 in the NF1 transcript. The combination of LR-PCR and RNA-seq allowed for high-resolution mapping of the transcriptome, facilitating the identification of deletions and splicing alterations that would be challenging to detect using whole exome sequencing alone. The use of minigene assays further validated the functional impact of the p. K329K variant, showing that the synonymous change led to partial loss of exon 9 when transfected into HEK293 cells, thereby confirming its role in abnormal splicing. Specifically, the mutant minigene consistently exhibited exon skipping, whereas the wild-type construct showed normal exon inclusion, underscoring the functional consequences of this synonymous variation.

Our study thus highlights the importance of considering synonymous variants as potential pathogenic mutations, particularly in complex disorders like NF1, where alternative splicing and regulatory mechanisms play a significant role in disease manifestation. The discovery that synonymous variations, traditionally considered benign, can alter splicing and contribute to disease adds a layer of complexity to genetic diagnostics and underscores the need for comprehensive functional assays. However, the precise molecular mechanisms by which the p. K329K variant influences the splicing machinery remain unclear. Further research focusing on functional assays to elucidate the interaction of this variant with SREs will be crucial for deepening our understanding of NF1 pathogenesis and could reveal novel therapeutic targets. Moreover, the findings in this study advocate for the broader application of RNA sequencing and LR-PCR in genetic studies to uncover cryptic splicing abnormalities that conventional sequencing may overlook (Ha et al., 2021; Koster et al., 2021; Florea, 2006; Yura et al., 2006; Verta and Jacobs, 2022; Manuel et al., 2023; Kim et al., 2007; Bush et al., 2017; Verta and Jacobs, 2022).

NF1 is caused by the NF1 gene, characterized by its large size, complex splicing patterns, and high variation rate (Messiaen et al., 2000). This usually leads to underreporting or misclassifying NF1 variations by traditional gene sequencing like WES (Messiaen and Wimmer, 2005). In our study, the synonymous variation c.987A>G (p.K329K) was identified by WES, which was subsequently confirmed to affect splicing by RNA-seq analysis. RNA-seq effectively reveals complex splicing patterns and regulatory mechanisms for deeper insights into the pathogenesis of NF1 (Ha et al., 2021; Koster et al., 2021). The use of minigene assays further validated these findings, providing a functional assessment of how this synonymous variant directly contributed to aberrant splicing by mimicking the splicing environment and confirming the presence of exon skipping. Specifically, we constructed a minigene containing the genomic region encompassing exon 9 and its flanking intronic sequences, then transfected it into HEK293 cells. The results showed a clear pattern where the mutant minigene resulted in skipping of exon 9, while the wild-type construct included exon 9 in the final transcript. This type of functional experiment provides direct evidence of how the synonymous variation influences splicing, confirming that the c.987A>G variant has a significant impact on the splicing process. However, given that our study included only two patients from a two-generation family, future studies would not only aim to expand the family cohort to further explore the relationship between this variant and its clinical manifestations but also focus on elucidating the precise molecular mechanisms by which this variant affects the function of splicing regulatory elements.

## 5 Conclusion

In conclusion, our study demonstrates that the synonymous variation c.987A>G (p.K329K) in the NF1 gene significantly impacts gene splicing, challenging the conventional view that synonymous variations are benign. The clinical variability observed in patients carrying the p. K329K variant highlights the complexity of genotype-phenotype relationships in NF1, emphasizing the importance of comprehensive molecular and functional analyses. Further research is needed to clarify the mechanisms underlying splicing disruption by synonymous variants, which may ultimately contribute to improved diagnosis and targeted therapies for NF1 and other similar genetic disorders.

## Data Availability

The original contributions presented in the study are publicly available. This data can be found here: [raw data link: https://pan.baidu.com/s/1aP2tPIhd6jBV3lkbqVmBNQ&pwd=u88y; WES gene report: https://pan.baidu.com/s/1An7UURqWywyZqxC3tegxdg?pwd=0409].
