# Supplementary figures and images for: A novel synonymous variant in the NF1 gene disrupting splicing contributes to neurofibromatosis pathogenesis
Source: Front Genet. 2025 May 9;16:1572487. doi: 10.3389/fgene.2025.1572487 (PMC12098384; doi:10.3389/fgene.2025.1572487)

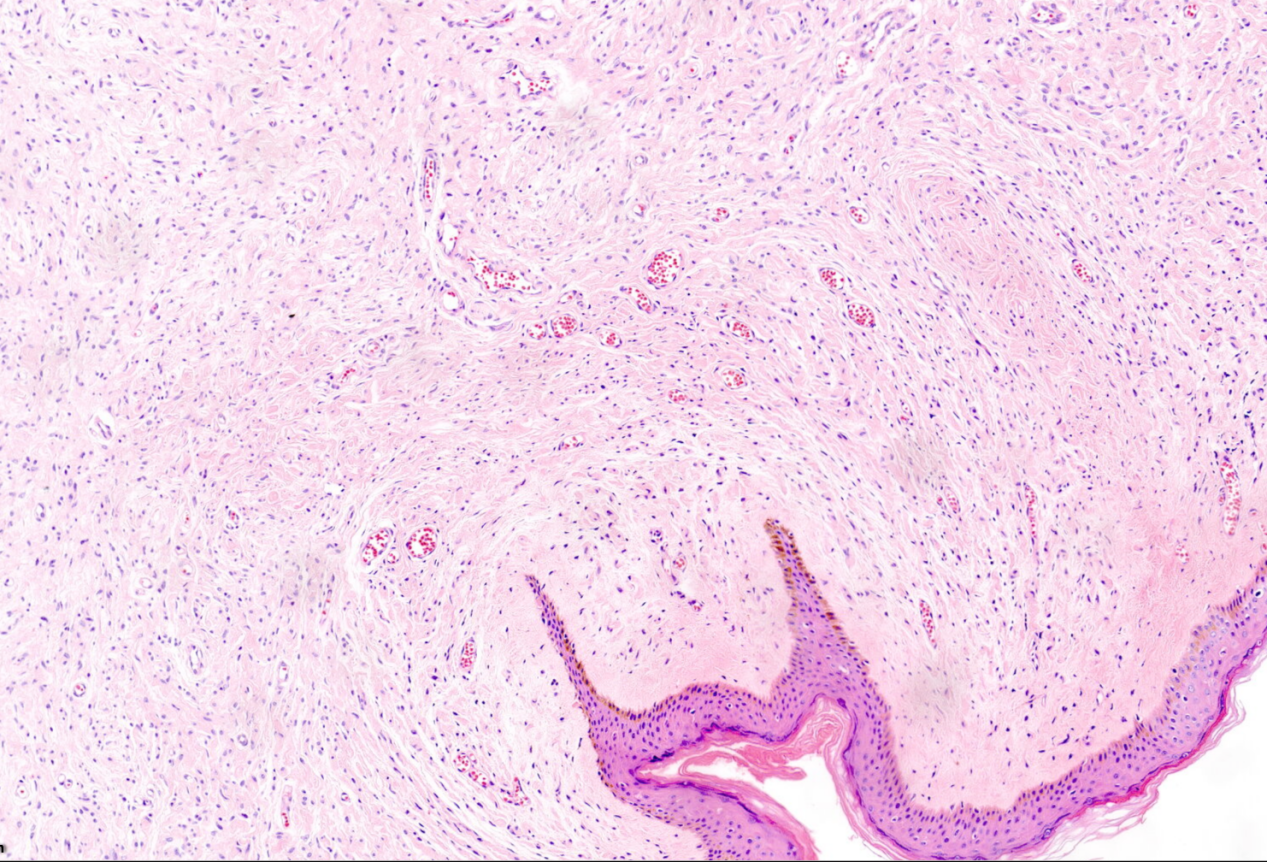

Supplement: Supplementary file 1 [file DataSheet1.zip › Supplementary Figure 1/Supplementary Figure 1A-blood vessels.tif]

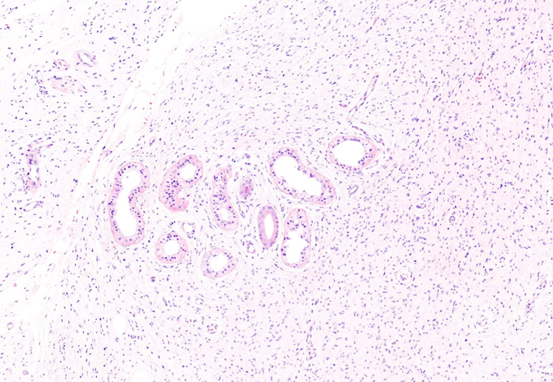

Supplement: Supplementary file 1 [file DataSheet1.zip › Supplementary Figure 1/Supplementary Figure 1A-sweat glands.tif]

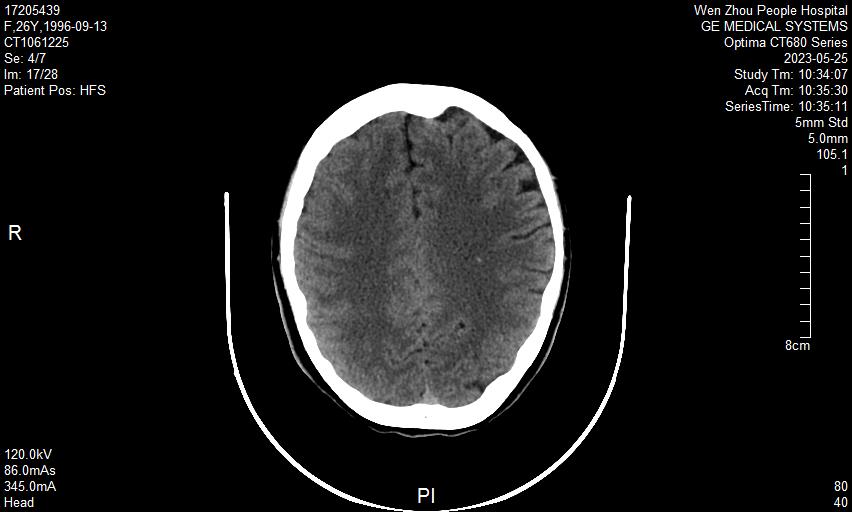

Supplement: Supplementary file 1 [file DataSheet1.zip › Supplementary Figure 1/Supplementary Figure 1B left.jpg]

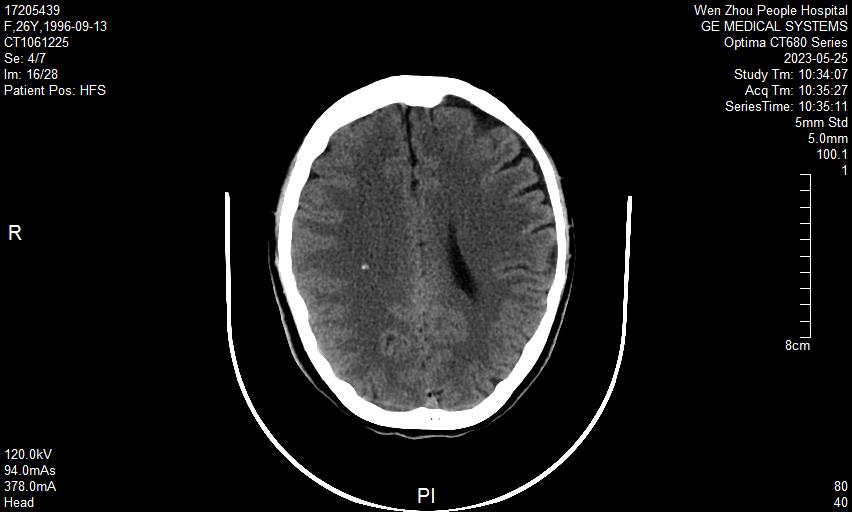

Supplement: Supplementary file 1 [file DataSheet1.zip › Supplementary Figure 1/Supplementary Figure 1B right.jpg]

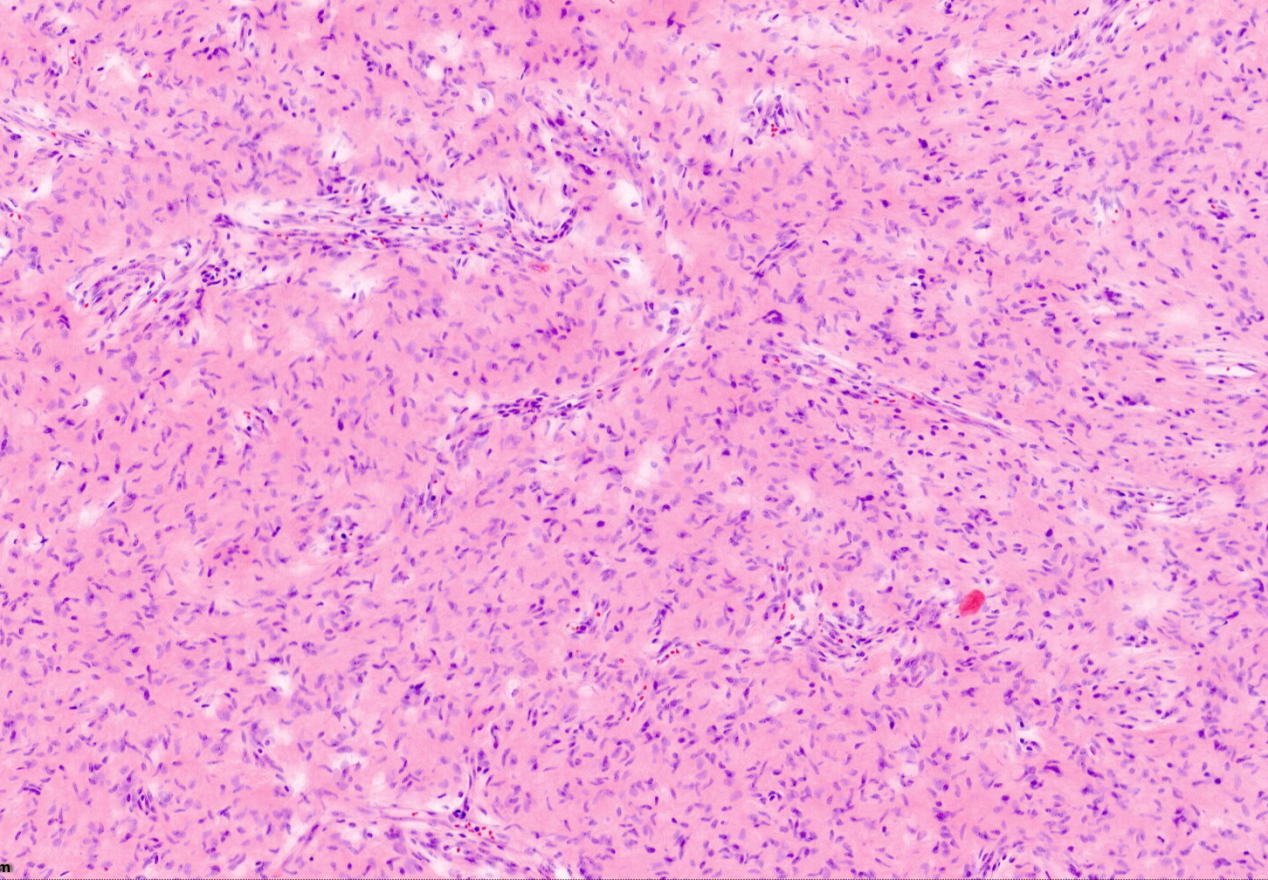

Supplement: Supplementary file 1 [file DataSheet1.zip › Supplementary Figure 1/Supplementary Figure 1C-blood vessels.tif]

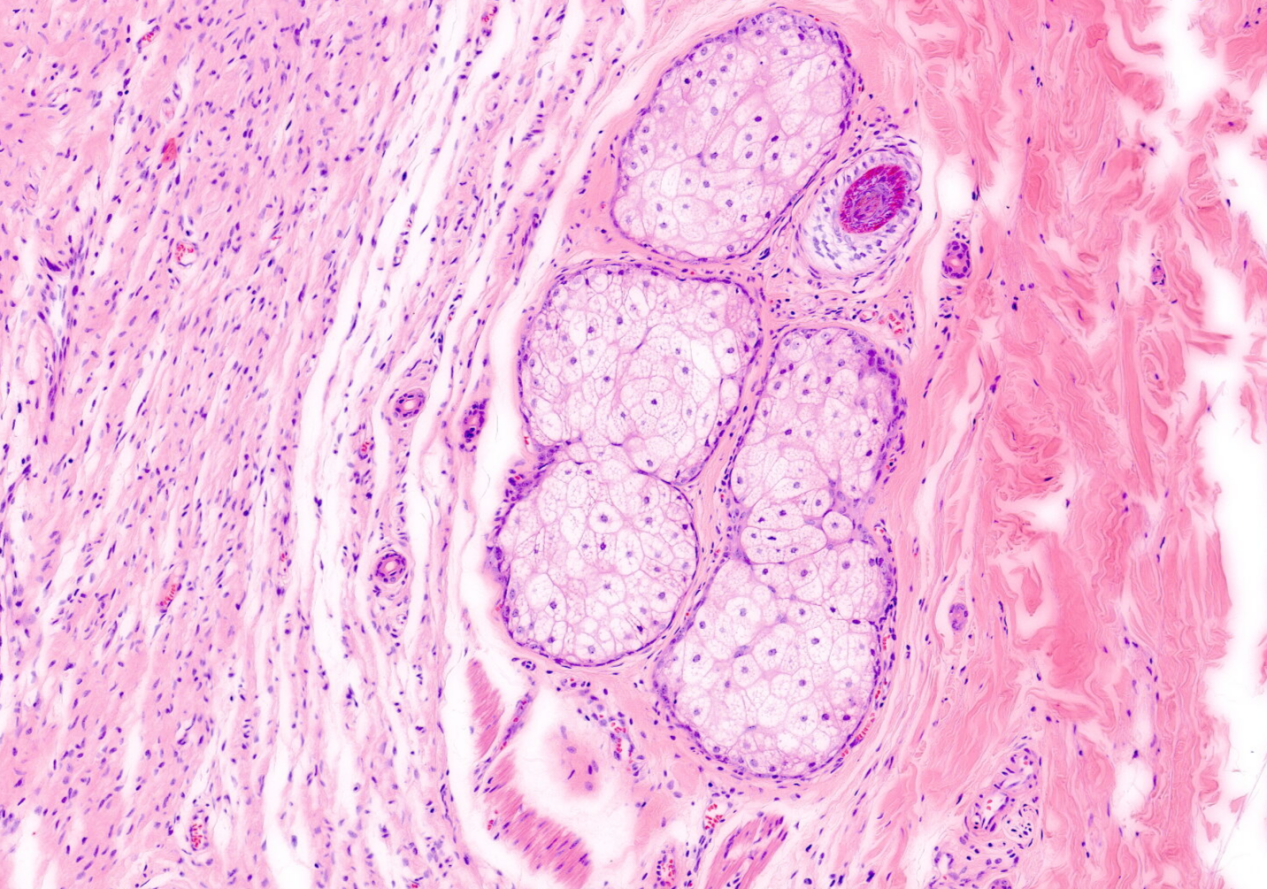

Supplement: Supplementary file 1 [file DataSheet1.zip › Supplementary Figure 1/Supplementary Figure 1C-sebaceous glands.tif]

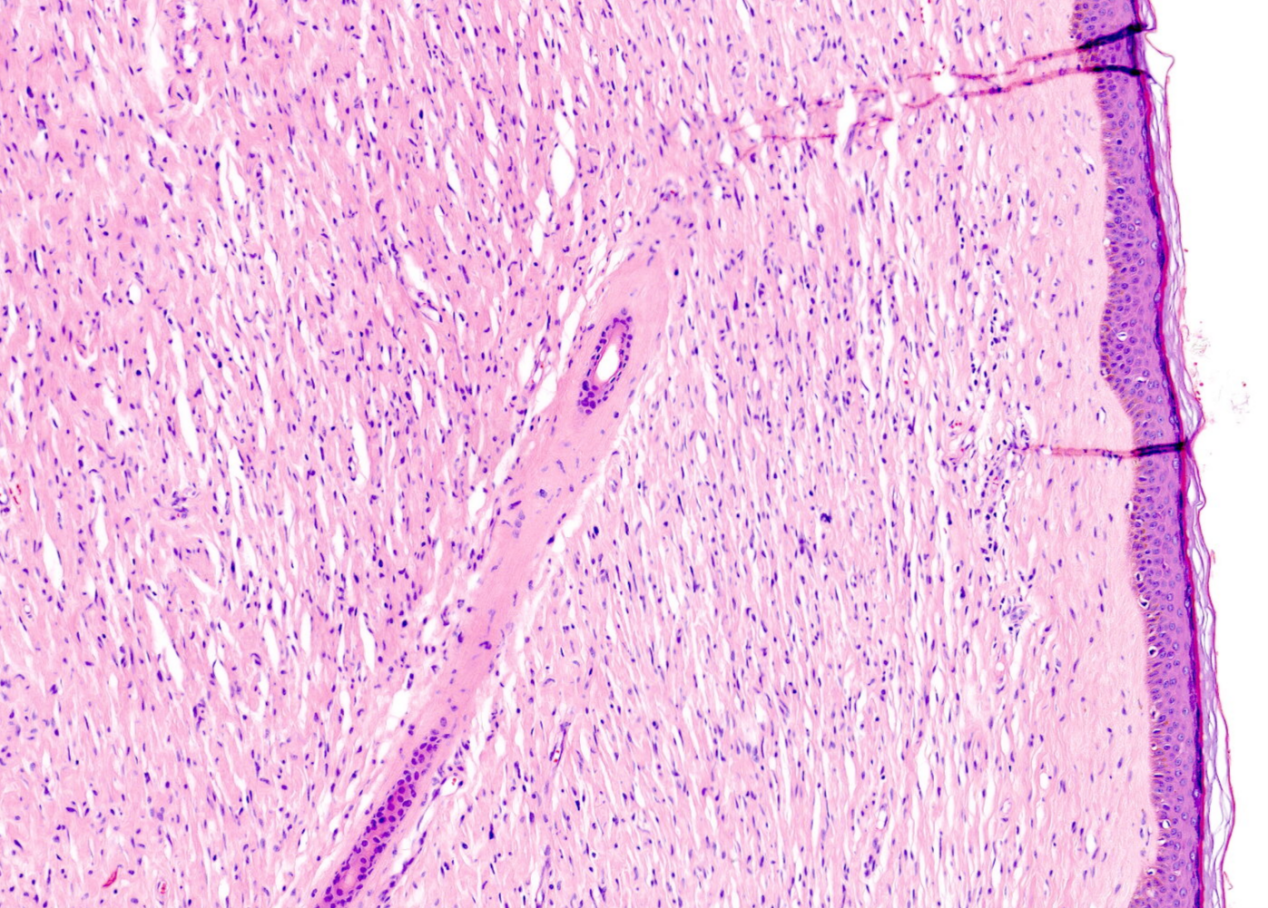

Supplement: Supplementary file 1 [file DataSheet1.zip › Supplementary Figure 1/Supplementary Figure 1C-Sweat ducts.tif]

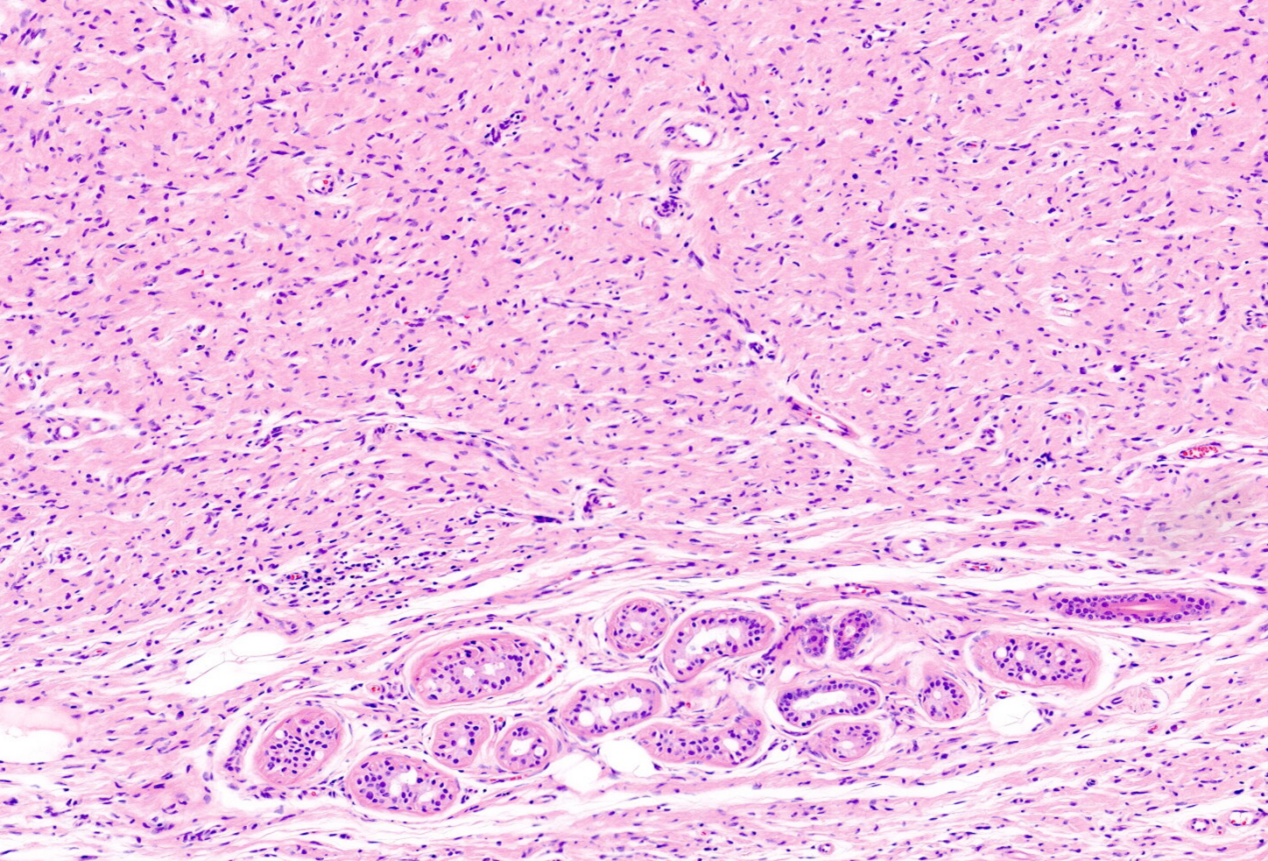

Supplement: Supplementary file 1 [file DataSheet1.zip › Supplementary Figure 1/Supplementary Figure 1C-Sweat glands.tif]

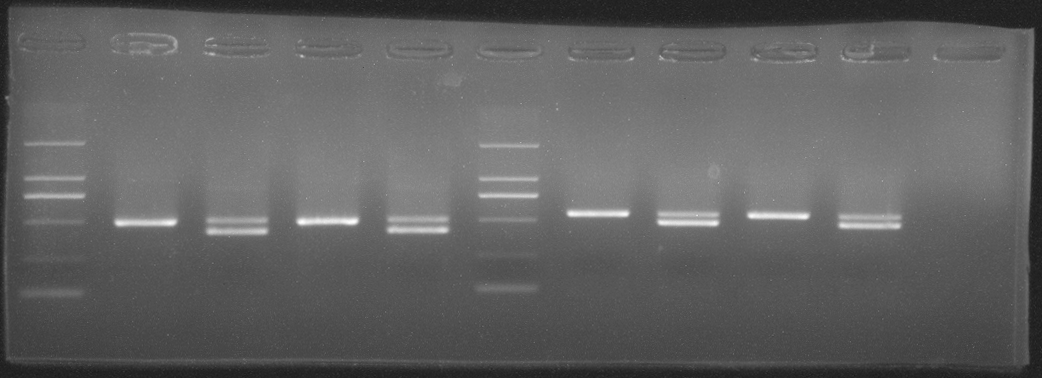

Supplement: Supplementary file 2 [file DataSheet2.zip › Supplementary Figure 2/Supplementary Figure 2B.png]
