# Supplementary material for: A novel synonymous variant in the NF1 gene disrupting splicing contributes to neurofibromatosis pathogenesis
Source: Front Genet. 2025 May 9;16:1572487. doi: 10.3389/fgene.2025.1572487 (PMC12098384; doi:10.3389/fgene.2025.1572487)
Supplement: Supplementary file 2 [file DataSheet2.zip › Supplementary Figure 2/Supplementary Figure 2A.pptx]

## Slide 1
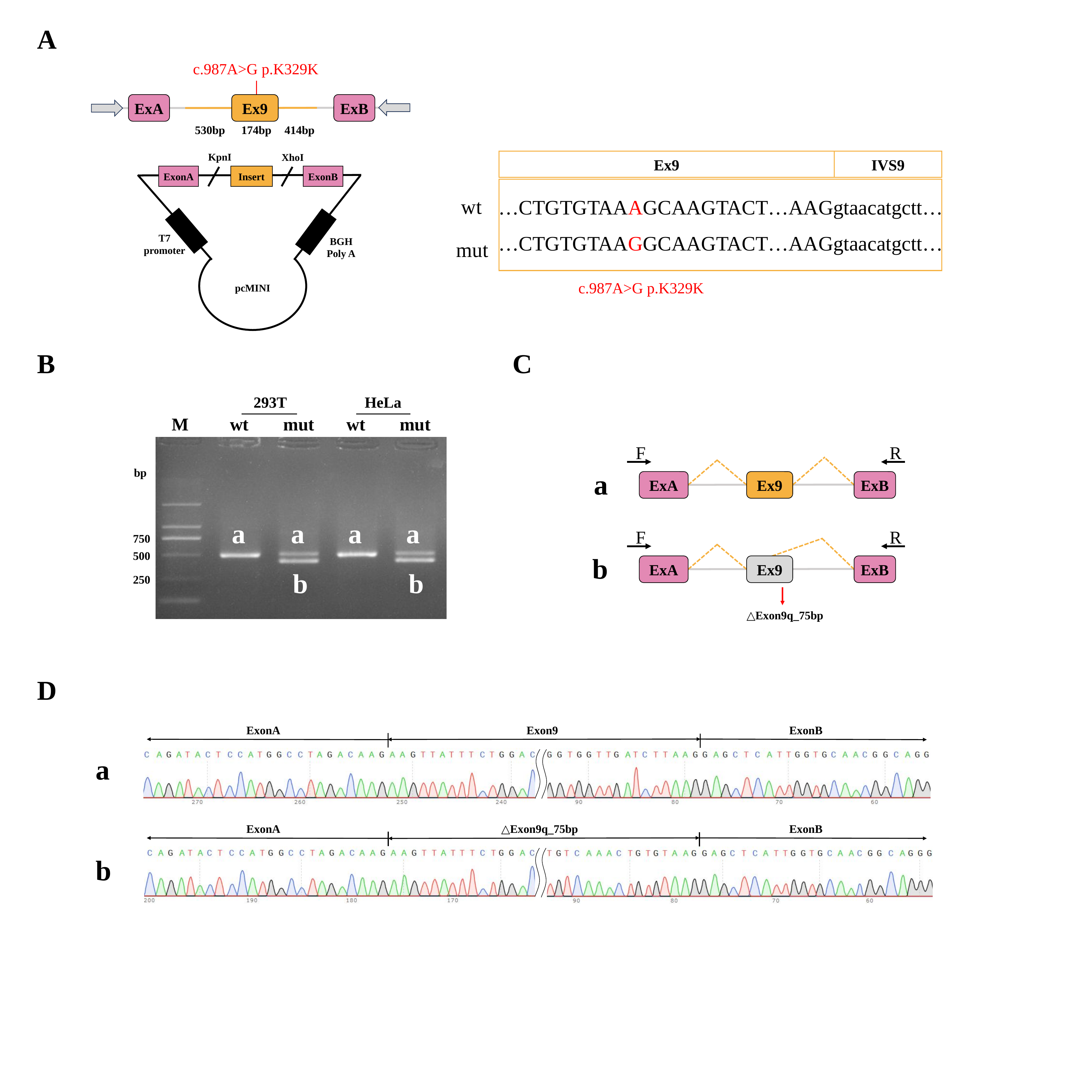

A
c.987A>G p.K329K
ExA
Ex9
ExB
530bp
174bp
414bp
KpnI
XhoI
IVS9
Ex9
ExonA
Insert
ExonB
wt
…CTGTGTAAAGCAAGTACT…AAGgtaacatgctt…
…CTGTGTAAGGCAAGTACT…AAGgtaacatgctt…
T7
promoter
BGH
Poly A
mut
c.987A>G p.K329K
pcMINI
B
C
293T
HeLa
M
wt
mut
wt
mut
F
R
bp
a
ExA
Ex9
ExB
a
a
a
a
F
R
750
500
b
ExA
Ex9
ExB
b
b
250
△Exon9q_75bp
D
ExonA
Exon9
ExonB
a
ExonA
△Exon9q_75bp
ExonB
b
